# Two-color Dye-swap DNA Microarray approach toward confident gene expression profiling in PMCAO mouse model for ischemia-related and PACAP38-influenced genes

**DOI:** 10.1016/j.gdata.2015.01.007

**Published:** 2015-01-22

**Authors:** Motohide Hori, Junko Shibato, Tomoya Nakamachi, Randeep Rakwal, Tetsuo Ogawa, Seiji Shioda, Satoshi Numazawa

**Affiliations:** a Division of Toxicology, Department of Pharmacology, Toxicology and Therapeutics, School of Pharmacy, Showa University, 1-5-8 Hatanodai, Shinagawa, Tokyo 142-8555, Japan; b Department of Anatomy, Showa University School of Medicine, 1-5-8 Hatanodai, Shinagawa, Tokyo 142-8555, Japan; c Laboratory of Exercise Biochemistry and Neuroendocrinology, Institute of Health and Sports Sciences, University of Tsukuba, Tsukuba, Ibaraki 305-8574, Japan; d Laboratory of Regulatory Biology, Graduate School of Science and Engineering, University of Toyama, Toyama, Toyama 930-8555, Japan; e Organization for Educational Initiatives, University of Tsukuba, 1-1-1 Tennoudai, Tsukuba 305-8577, Ibaraki, Japan

**Keywords:** Permanent middle cerebral artery occlusion, Brain ischemia, Infract core, Penumbra, Neuropeptide

## Abstract

Toward twin goals of identifying molecular factors in brain injured by ischemic stroke, and the effects of neuropeptide pituitary adenylate-cyclase activating polypeptide (PACAP) on the ischemic brain, we have established the permanent middle cerebral artery occlusion (PMCAO) mouse model and utilized the Agilent mouse whole genome 4 × 44 K DNA chip. PACAP38 (1 pmol) injection was given intracerebroventrically in comparison to a control saline (0.9% NaCl) injection, to screen genes responsive to PACAP38. Two sets of tissues were prepared, whole hemispheres (ischemic and non-ischemic) and infract core and penumbra regions at 6 and 24 h. In this study, we have detailed the experimental design and protocol used therein and explained the quality controls for the use of total RNA in the downstream DNA microarray experiment utilizing a two-color dye-swap approach for stringent and confident gene identification published in a series of papers by Hori and coworkers (Hori et al., 2012–2015).

**Table:** 

Specifications	
Organism/cell line/tissue	*Mus musculus*
Strains(s)	Brain (hemisphere/ischemic regions/non-ischemic regions)
Sequencer or array type	Agilent whole mouse genome microarray G4122F
Data format	Raw data: TXT file, Normalized data: TXT
Experimental factors	Cerebral ischemia; permanent middle cerebral artery occlusion; neurological grade; ipsilateral hemisphere, non-injected; contralateral hemisphere, injected with saline or PACAP38; time of ischemia; dissection of brains; infract core and penumbra regions; total RNA extraction; RT-PCR
Consent	Animal care and experimental procedures approved by Institutional Animal Care and Use Committee of Showa University (School of Medicine), Tokyo, Japan.

## Direct link to deposited data

Deposited data can be found here: GSE28201 (http://www.ncbi.nlm.nih.gov/geo/query/acc.cgi?acc=GSE28201), GSE37565 (http://www.ncbi.nlm.nih.gov/geo/query/acc.cgi?acc=GSE37565), and GSE62884 (http://www.ncbi.nlm.nih.gov/geo/query/acc.cgi?acc=GSE62884).

**Data, experimental design, materials and methods**

### Generation of the permanent middle cerebral artery occlusion model (PMCAO)

Brain ischemia, also termed cerebral ischemia or ischemic stroke, is a major health problem worldwide [1]. Extremely debilitating to the brain tissues, it is the third most common cause of death after heart attack and cancer. The permanent middle cerebral artery occlusion (PMCAO) model was used over the transient MCAO, due to the avoidance of reperfusion injury in our research model aimed at providing new insight into the PACAP38 neuroprotective effects in the mouse brain [2], [3], [4], [5], [6]. PMCAO was performed on male mice (C57/BL6J), and the results of the procedure were monitored by dissecting whole brains and visualizing the extent of ischemia. More than 80% of the mice survived the PMCAO procedure. Post-PMCAO, the effect of neuropeptide pituitary adenylate-cyclase activating polypeptide 38 (PACAP38) treatment (injection was performed intracerebroventrically) was examined in mouse; saline injection was used as a control [2], [3], [4], [5], [6]. The aim was two-fold: first, to provide a detailed ischemia-related transcriptome in mouse model, and second, was to explore the neuroprotective effects of PACAP38 via gene inventories specific to PACAP38 effect and screening out the molecular factors and networks/pathways being activated therein. The reason for using, DNA microarray technology – one of the breakthrough technologies in experimental molecular biology – was that it allows us to quickly observe the expression of tens of thousands of genes in parallel in a single experiment. To be emphasized, this seemingly “quick” data is the product of a good experiment design, planning, sampling of tissues and proper storage, and quality control of the total RNA prior to the use of the microarray chip itself.

In order to achieve the above objectives, two basic approaches were taken that involved using hemispheres, ipsilateral containing the ischemic or infract core (called hereafter IC), penumbra (called thereafter P) and non-ischemic regions, and contralateral (non-ischemic) in the first set of experiments with or without PACAP38 treatment, and the second involving dissection of specific regions in the ischemic hemisphere, namely the IC and the P. The model is described in [2], [3], [4], [5], [6] and ischemia was confirmed by not only visual observation of the dissected brains but also by triphenyltetrazolium chloride (TTC) staining [7].

The gene expression data from these studies on hemispheres and specific regions (IC and P) were deposited in the Gene Expression Omnibus (GEO) database under accession numbers GSE28201 (http://www.ncbi.nlm.nih.gov/geo/query/acc.cgi?acc=GSE28201) [2], [3], GSE37565 (http://www.ncbi.nlm.nih.gov/geo/query/acc.cgi?acc=GSE37565) [4], and GSE62884 (http://www.ncbi.nlm.nih.gov/geo/query/acc.cgi?acc=GSE62884) [5], [6].

## Sampling sites and regimes, quality control of total RNA, and semi-quantitative RT-PCR and gene expression data

The mouse exhibiting neurological grades G1 and G2 were used for all experiments (Table 1) post PMCAO or after PACAP38 test solution injection over a saline control [2], [3], [4], [5], [6]. We followed all animal care and experimental procedures under approval by the Institutional Animal Care and Use Committee of Showa University (School of Medicine), Tokyo, Japan. Two time points were selected, 6 h and 24 h post-PMCAO [2], [3], [4], [5], [6] for sampling of the hemispheres and specific regions (IC and P), which were immersed immediately post-dissection in liquid nitrogen and thereafter transferred to a − 80 °C deep freezer. The samples were individually ground in liquid nitrogen to prepare very fine powders for subsequent downstream gene expression analyses [2], [3], [4], [5], [6] and proteomic analysis as required [4]. Prior to each DNA microarray analysis, the total RNA, extracted using an optimized protocol based on the QIAGEN RNeasy Mini Kit (QIAGEN, Germantown, MD, USA) quantity and quality was measured spectrophotometrically with NanoDrop (Thermo Scientific, Wilmington, DE, USA) and re-confirmed using formaldehyde-agarose gel electrophoresis [2], [3], [4], [5], [6], [8], [9]. Briefly, the obtained total RNA quality measurements revealed good quality as demonstrated by the A_260/280_ values > 1.8, A_260/230_ > 1.8; in our experiments, we always aim for a higher value of more than 2.0 to 2.3 [2], [3], [4], [5], [6]. Furthermore, the total RNA was obtained in optimum quantity of > 300 ng/μl. Following the visualization for confirming pre-experimental integrity of the total RNA subunits by gel electrophoresis, an additional step comprised of cDNA synthesis was used for examining the quality of the synthesized cDNA by determining the expression of genes *glyceraldehyde 3-phosphate dehydrogenase* (*Gapdh*) and *beta-actin* as positive controls. This was done by RT-PCR (AffinityScript QPCR cDNA Synthesis Kit; Stratagene, Agilent Technologies, La Jolla, CA, USA and Emerald Amp PCR Master Mix; TaKaRa Shuzo, Shiga, Japan) on a Thermal Cycler (Applied Biosystems, Tokyo, Japan). Moreover, only gene-specific primers were used and whose sequences are shown in the articles [2], [3], [4], [5], [6]. Post-electrophoresis, the PCR products were visualized and quantified using a ChemiDoc XRS + imaging system (Bio-Rad Laboratories, Inc., Hercules, CA, USA). All the steps followed in sequence provided confidence that we had the best quality total RNA for use in the DNA microarray chip. As a last step, the Agilent mouse whole genome 4 × 44 K (G4122F) DNA slide (composed of 4 chips on one slide) was used for the microarray analysis, which was performed according to the Agilent instructions (Agilent Technologies, Santa Clara, CA, USA) and detailed in our publications [2], [3], [4], [5], [6], [8], [9]. As also mentioned below, our DNA microarray analysis experiment is based on the two-color dye-swap approach [10]. Microarray and sample annotation data were deposited in the Gene Expression Omnibus (GEO) database under accession numbers GSE28201 [2], [3], GSE37565 [4], and GSE62884 [5], [6].

## Gene expression analysis comparison between RT-PCR and DNA microarray experiment design (dye-swap) using positive controls

**Fig. 1:**
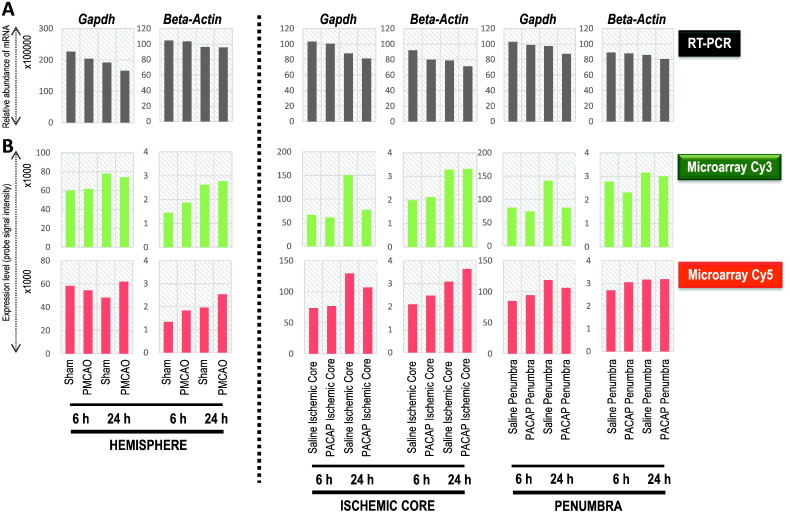
Expression of positive control genes, *glyceraldehyde 3-phosphate dehydrogenase* (*Gapdh*) and *beta-actin*, under RT-PCR and DNA microarray experiments. A, RT-PCR results presented graphically for *Gapdh* and *beta-actin* mRNA relative abundance. Each gene was analyzed at least twice as independent PCR reactions, and the representative data post-PCR band quantification is presented. B, The expression of these two genes is given by the expressed level of probe signal intensity in Cy3 and Cy5 labels, following the Feature Extraction step in the Agilent microarray analysis software. Total RNA was extracted from the whole hemisphere (hemisphere) and ischemic core and penumbra in the ipsilateral (right) hemisphere at 6 and 24 h. PACAP38 was the treatment and saline served as the control (sham). Semi-quantitative RT-PCR and DNA microarray analysis was performed as discussed in Sampling sites and regimes, quality control of total RNA, and semi-quantitative RT-PCR and gene expression data.

**Fig. 2:**
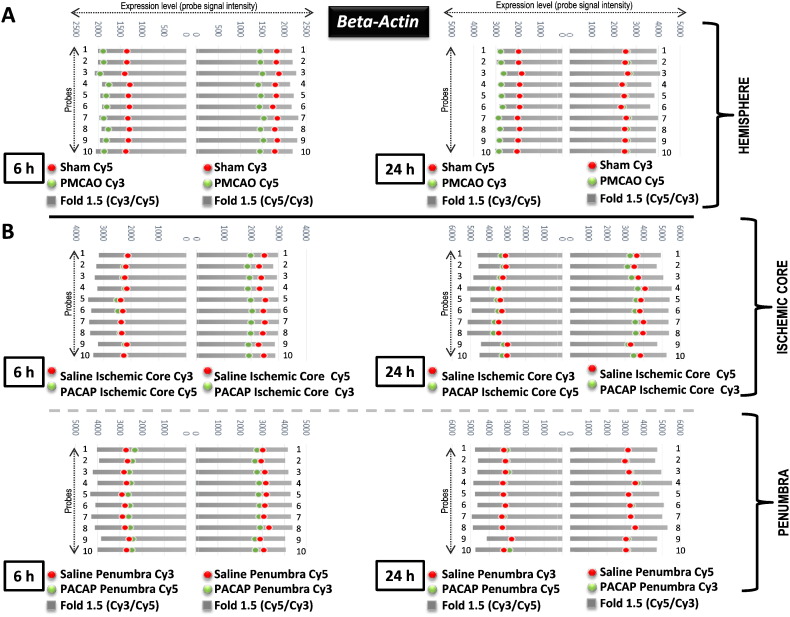
Expression level of the *beta-actin* gene, by expressed level of probe signal intensity in Cy3 and Cy5 labels under DNA microarray experiment in the whole hemisphere (hemisphere) and ischemic core and penumbra in the ipsilateral (right) hemisphere at 6 and 24 h under PACAP38 and saline (sham) treatments. Ten probes for the *beta-actin* gene are plotted for both labels, Cy3 and Cy5 under Sham, PMCAO in hemisphere and saline and PACAP38 treatments for both ischemic core and penumbra for specific regions microarray experiments using a total of 2 chips each. DNA microarray was performed as in Fig. 1.

The first quality control step presented itself in examining the expression of two genes, *Gapdh* and *beta-actin* normally used as house-keeping genes or positive control genes by RT-PCR for all samples used in the study; we prefer to use the latter terminology [11]. Fig. 1 compares the expression of these two control genes using the RT-PCR analysis (A) and the dye-swap based DNA microarray experiment (B) for each sample, control and treatment in whole hemisphere and specific regions of IC and P, at both 6 and 24 h time points. To note, for RT-PCR each gene expression (*Gapdh* and *beta-actin*) analysis is performed at least twice as independent PCR reactions (at different cycles for avoiding taking the data at a plateau) and electrophoresis on gel for each cDNA generated/sample [2], [3], [4], [5], [6], and a representative data is presented in Fig. 1A. Similarly, the probe signal intensity level is for a single probe each for the two genes on the DNA microarray chip (Fig. 1B). Comparing these representative data, some variation of gene expression can be seen in the groups at 6 or 24 h time points, however, the expression of these genes did not dramatically vary, rather showed almost similar expression levels between the RT-PCR based analysis and the microarray experiment. In particular, similar expression levels (probe signal intensity) were seen with Cy3 and Cy5 labels. Thus the expression of positive control genes normally used in our research, and other studies, in general, are quite stable over all sample conditions and times. Based on this analysis, not only the stable expression of some positive control genes but also the quality of synthesized cDNA was established. Moreover, these gene expressions were also found to be stable under the employed DNA microarray approach, namely dual-color and dye-swap. Furthermore, to check if the expression level on the microarray slide is similar across all probes plotted on the microarray chip, we examined the Cy3 and Cy5 label associated expression for all 10 probes for the *beta-actin* gene as an example, in the whole hemisphere and IC and P regions at both 6 h and 24 h time points (Fig. 2). Results showed a good correlation between the Cy3 and Cy5 label expression points across the 1.5-fold change ratio. This further confirms the data presented in Fig. 1, indicating that the gene expression for positive controls is stable across samples and also individual probes used in a DNA microarray design, and that can be compared with the RT-PCR-based analysis method.

## Hybridization, normalization, and scatter plot distribution of intensity values

**Fig. 3:**
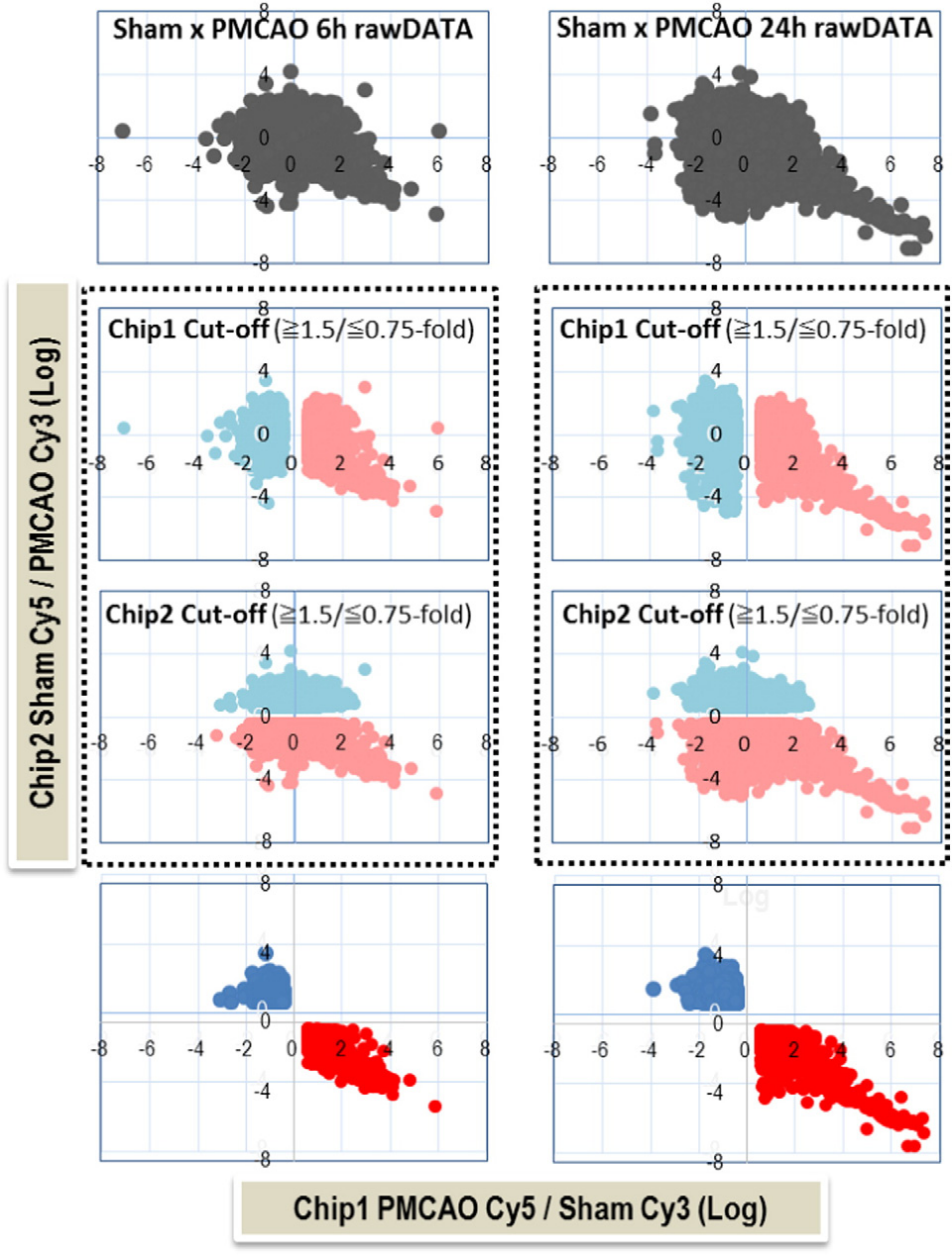
Intensity scatter plot of the two colors/chips. Upper-most two graphs show the raw data at 6 and 24 h, Sham × PMCAO. Each spot on the graph refers to a single gene probe. The genes plotted on each graph represent all the genes on the chip, chip 1 and chip 2, which were used for dye-swap analysis as shown in the center four graphs (in dotted-line boxes). The cut-off for chip 1 and chip 2 has an up-regulated and down-regulated fold ratio of greater than or equal to 1.5 (genes indicated by pink color) and less than or equal to 0.75 (genes indicated by sky blue color); the blank vertical (for chip 1) and horizontal (chip 2) spaces between the sky blue and pink colors represent the no change in expression genes. For the dye-swap to take place (selection), the genes showing an up-regulated fold-change in chip 1 (pink) need to show a corresponding down-regulated value in chip 2 (blue) and vice versa for the down-regulations (pink). Then, the lower-most two graphs show the dye-swap scatter plots which reveal reliable/confident gene expression selected (common up-regulated ≧ 1.5-fold are in red; common down-regulated ≦ 0.75 are in blue) based on the chip 1 and chip 2 data analyses as described above. Genes with similar expression are on the diagonal; log-transformed expression values are provided.

Fluorescently labeled targets of control (sham/saline) as well as treatments (PACAP38) were hybridized to the same microarray slide with 60-mer probes. Selection of differentially expressed genes was performed in a way that a gene up-regulated in chip 1 (Cy3 label for sham and Cy5 label for PMCAO) was down-regulated in chip 2 (Cy5 label for sham and Cy3 label for PMCAO) (Fig. 3). A similar data were presented for the PACAP38 versus sham (saline) treatments for the IC and P regions (Supplementary figure 1, Supplementary figure 2). The genes that were selected thus had a higher confidence, but were less than the numbers obtained by using just one dye orientation. As also elegantly explained by Dombkowski and co-workers in 2004, dye orientation has a significant influence on the measurements and inferences of differential gene expression most probably due to the labeling method, and further suggesting that until dye bias effects can be explained the dye-swap approach is important in microarray reference designs [10]. This still stands true today. Hybridization and wash processes were performed according to the manufacturer's instructions, and hybridized microarrays were scanned using an Agilent Microarray scanner G2565BA or G2505C (Agilent Technologies). For the detection of significant differentially expressed genes between control and treated samples each slide image was processed by Agilent Feature Extraction software (version 9.5.3.1 or 11.0.1.1, Agilent Technologies). The Feature Extraction algorithms have been developed to reduce systematic errors arising from labeling bias, irregular feature morphologies, mismatched sample concentrations and cross hybridizations [12]. By this program, the Cy3 and Cy5 signal intensities of whole probes are measured. Dye-bias tends to be signal intensity dependent therefore the software selects probes using a set by rank consistency filter for dye-normalization. Said normalization is performed by LOWESS (locally weighted linear regression) which calculates the log ratio of dye-normalized Cy3- and Cy5-signals, as well as the final error of log ratio. The statistical significance of the log ratio values obtained for individual microarray probes is calculated using the “most conservative error model” default function of the Feature Extraction software, by which both a propagated and a universal error model are evaluated, and the higher (more conservative estimate of error) p-value of two error models is reported [[13], [14]; Agilent Technologies]. The propagated error model measures the error on the log ratio by propagating the pixel-level error from calculations made in the analysis (i.e., raw signal and background subtraction), whereas the universal error model measures the expected error between the red and green channels using the additive and multiplicative errors (Agilent Technologies). In these analyses, the threshold of significant differentially expressed genes was < 0.01 (for the confidence that the feature was not differentially expressed). Additionally, the erroneous data generated due to artifacts were eliminated before data analysis by the software.

## Validation of DNA microarray data using RT-PCR-based analysis of interleukin 6 (*Il6*) and brain-derived neurotrophic factor (*Bdnf*) gene expressions

**Fig. 4:**
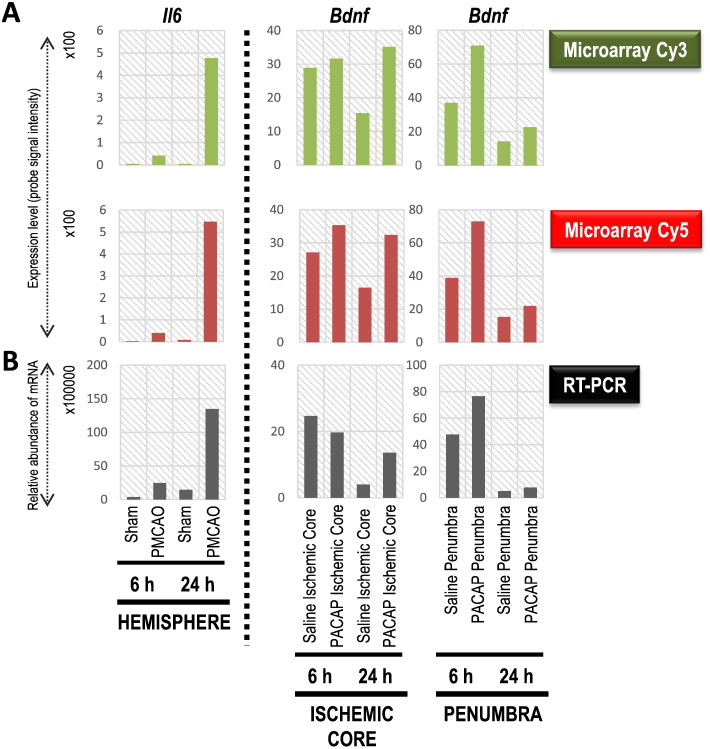
Expression of two differentially expressed genes, *interleukin 6* (*Il6*) and *brain-derived neurotrophic factor (Bdnf)*, under DNA microarray experiment and subsequent validation by RT-PCR. A, Expression of *Il6* and *Bdnf* genes by expressed level of probe signal intensity with Cy3 and Cy5 labels. B, RT-PCR results are presented graphically for these two genes mRNA relative abundance. Total RNA was extracted from the whole hemisphere (hemisphere) and ischemic core and penumbra in the ipsilateral (right) hemisphere at 6 and 24 h. PACAP38 was the treatment and saline served as the control (sham). DNA microarray analysis and RT-PCR were performed as in Fig. 1.

Two differentially expressed genes, namely *Il6* and *Bdnf*, as examples in the PMCAO (*Il6*) and PACAP38 influenced (*Bdnf*) experiments were examined and compared for their expression levels between the microarray experiment (Fig. 4 A) and RT-PCR (Fig. 4 B). A very good correlation was seen between these two experiments for both *Il6* and *Bdnf*. A slight discrepancy was seen the case of the *Bdnf* gene, in the ischemic core region sample at 6 h, where a slight increase in expression under the microarray experiment (cut-off of greater than 1.5 fold) could not be confirmed by the RT-PCR analysis; it may be dependent on the probe design used for RT-PCR or the only slight induction seen at 6 h under the microarray data. Nevertheless, from this analysis as also in Fig. 1 for positive control genes, a good correlation between the Cy3 and Cy5 values from the microarray experiment could be seen. These results not only show the importance of a validation step using an independent method of gene expression analysis but also show that the dye-swap approach-based screening of gene expressions is a reliable indicator of the effect of the treatment being performed.

## Discussion

We described here unique datasets of i) ischemia-related genes and ii) genes potentially related to the neuroprotective effects of the neuropeptide PACAP38 from the brains of PMCAO model mouse. Each of the three datasets is composed of genome-wide gene expression measured using the Agilent Gene Expression Microarray platform using the dye-swap approach (Agilent Technologies). We showed that the brain response to ischemia can be evaluated at the level of the gene, genome-wide, and presented the first ischemia-related transcriptome analysis of the mouse brain in a series of studies using the Agilent DNA microarray chip using both ipsilateral and contralateral hemispheres. Furthermore, as our primary interest is to clarify the role of the neuropeptide PACAP38 in protecting or as a therapy for brain ischemia, we have also comprehensively analyzed the brain responses in ischemic brain after treatment (PACAP38) over saline (sham). It is our idea that these large numbers of gene inventories obtained will provide a strong basis for further research into not only understanding the ischemic brain but also in pinpointing gene candidates potentially involved in mediating the neuroprotective effect of PACAP38.

The following are the supplementary data related to this article.

Supplementary figure 1 Intensity scatter plot of the two colors/chips for the ischemic core (IC) experiment under PACAP38 treatment at 6 and 24 h time points.

Supplementary figure 2 Intensity scatter plot of the two colors/chips for the penumbra (P) experiment under PACAP38 treatment at 6 and 24 h time points.

## Figures and Tables

**Table 1:** The permanent middle cerebral artery occlusion (PMCAO) experimental mouse model and the effect of PMCAO (ischemia), PACAP38 and saline treatments. Mouse were selected randomly based on neurological grade (NG) 1 and 2 but not 0 and 3 for sampling.

Mouse tail no.	Body weight before operation (g)	Neurological grade (NG)
*PMCAO injected normal saline, and decapitated after 6 h*		
1 to 14	26.78, 26.54, 24.4, 30.64, 30.58, 25.93, 22.87, 22.53, 25.97, 23.47, 24.43, 23.15, 22.21, 23.00	2, 1, 2, 1, 0, 2, 2, 3, 2, 2, 0, 2, 0, 2

*PMCAO injected normal saline, and decapitated after 24 h*		
1 to 17	26.44, 26.93, 26.65, 24.64, 25.85, 25.98, 26.71, 24.15, 22.55, 22.82, 23.64, 22.51, 23.05, 24.44, 23.87, 23.54, 23.60	2, 2^a^, 1, 3, 1^a^, 1, 1, 1, 0, 1, 2, 0, 3, 2, 2, 3, 3

*PMCAO injected PACAP38, and decapitated after 6 h*		
1 to 6	23.78, 24.72, 24.33, 24.57, 25.17, 23.89	2, 1, 2, 1, 1, 2

*PMCAO injected PACAP38, and decapitated after 24 h*		
1 to 7	25.92, 26.84, 26.52, 25.53, 23.00, 23.29, 23.91	1, 1, 1, 1, 2, 3, 2

*Sham injected normal saline, and decapitated after 6 h*		
1 to 5	25.70, 24.27, 28.19, 26.96, 27.03	0, 0, 0, 0, 0

*Sham injected normal saline, and decapitated after 24 h*		
1 to 5	24.15, 25.17, 25.99, 25.28, 25.90	0, 0, 0, 0, 0

*Sham injected PACAP38, and decapitated after 6 h*		
1 to 5	25.06, 25.93, 25.82, 24.88, 26.34	0, 0, 0, 0, 0

*Sham injected PACAP38, and decapitated after 24 h*		
1 to 5	23.93, 24.48, 25.40, 24.04, 25.48	0, 0, 0, 0, 0

a Indicates dead mouse.
