# Supplementary material for: Two-color Dye-swap DNA Microarray approach toward confident gene expression profiling in PMCAO mouse model for ischemia-related and PACAP38-influenced genes
Source: Genom Data. 2015 Jan 22;3:148–54. doi: 10.1016/j.gdata.2015.01.007 (PMC4535747; doi:10.1016/j.gdata.2015.01.007)
Supplement: Supplementary figure 1 — Intensity scatter plot of the two colors/chips for the ischemic core (IC) experiment under PACAP38 treatment at 6 and 24 h time points. [file mmc1.pptx]

## Slide 1
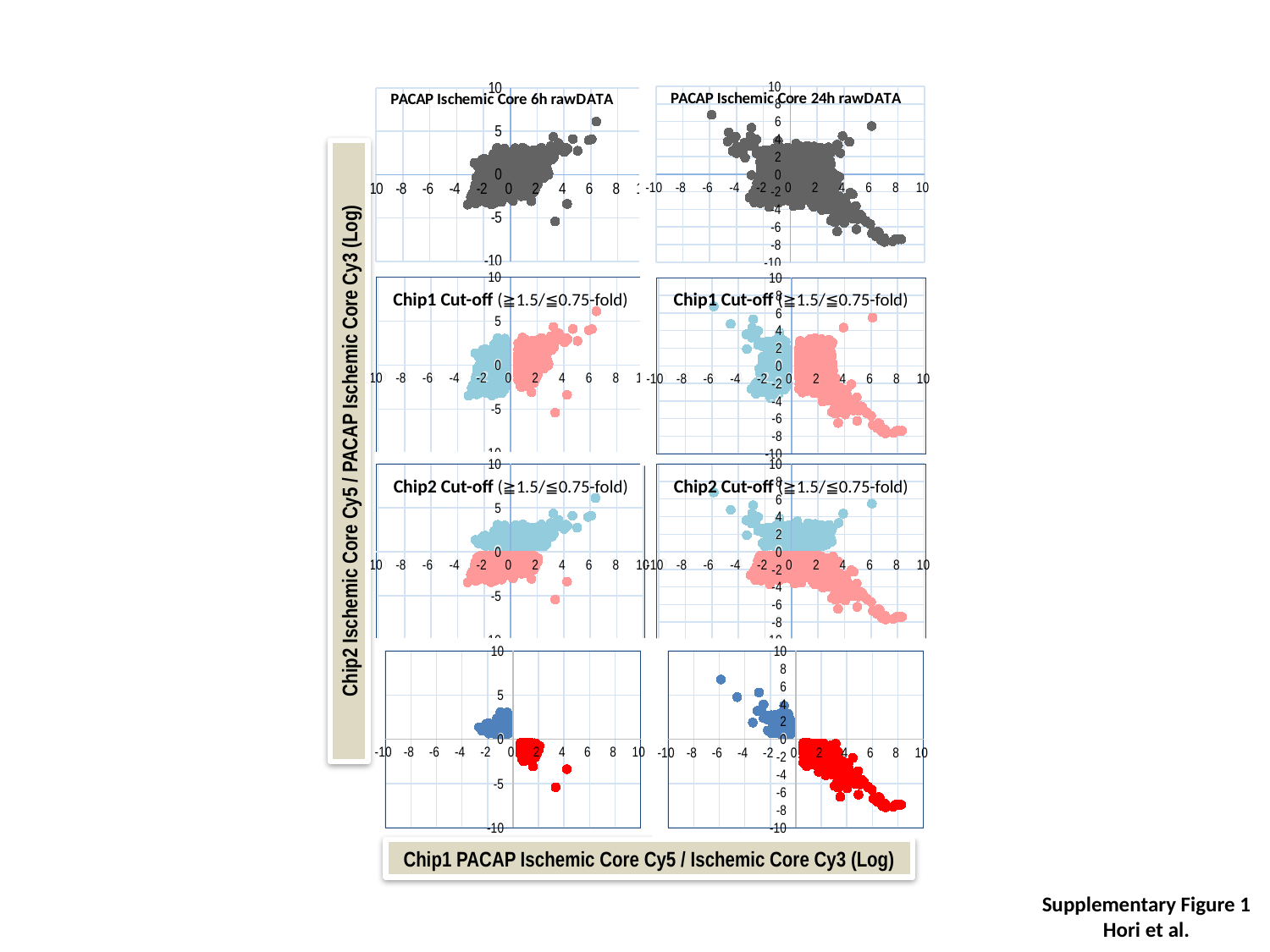

### Chart
| Category | LOG2 | | |
|---|---|---|---|
### Chart
| Category | LOG2 | | |
|---|---|---|---|
### Chart
| Category | -1 |
|---|---|
### Chart
| Category | 1 |
|---|---|
### Chart
| Category | 0 |
|---|---|
### Chart
| Category | 0 |
|---|---|Chip2 Ischemic Core Cy5 / PACAP Ischemic Core Cy3 (Log)
### Chart
| Category | LOG2 |
|---|---|
### Chart
| Category | LOG2 |
|---|---|
### Chart
| Category | LOG2 |
|---|---|
### Chart
| Category | LOG2 |
|---|---|
### Chart
| Category | LOG2 |
|---|---|
### Chart
| Category | -1 |
|---|---|
### Chart
| Category | LOG2 |
|---|---|
### Chart
| Category | -1 |
|---|---|Chip1 PACAP Ischemic Core Cy5 / Ischemic Core Cy3 (Log)
Chip1 Cut-off (≧1.5/≦0.75-fold)
Chip1 Cut-off (≧1.5/≦0.75-fold)
Chip2 Cut-off (≧1.5/≦0.75-fold)
Chip2 Cut-off (≧1.5/≦0.75-fold)
Supplementary Figure 1
Hori et al.
